# Impact of khat price increases on consumption behavior – price elasticity analysis

**DOI:** 10.1186/s13011-019-0208-3

**Published:** 2019-05-14

**Authors:** Maged El-Setouhy, Rashad Alsanosy, Anwar M. Makeen, Khalid Yaser Ghailan, Abdullah Alsharqi, Kamaludin Ahmed Sheikh

**Affiliations:** 1 Department of Family and Community Medicine, Faculty of Medicine, Jazan University, Jazan, Kingdom of Saudi Arabia; 2 Substance Abuse Research Center (SARC), Jazan University, Jazan, Kingdom of Saudi Arabia; 3 Department of Epidemiology, Faculty of Public Health &Tropical Medicine, Jazan University, Jazan, Kingdom of Saudi Arabia; 4 Department of Community, Occupational and Environmental Medicine, Faculty of Medicine, Ain Shams University, Cairo, Egypt; 5 Psychcare Clinics, Riyadh, Saudi Arabia

**Keywords:** Khat chewing index, Price elasticity of demand, Law enforcement strategies, One-way repeated measures ANOVA

## Abstract

**Background:**

The long border of Saudi Arabia with Yemen is the primary route for khat entry to the Kingdom. As of April 2015, the government of SA tightened the border, making it more difficult to import khat into the country. As a result, local user prices of khat probably increased due in part to higher supply costs and perhaps lower quantities. One anti-drug strategy is to increase consumption cost by increasing the price of supply. We aim in this study to measure the responsiveness of khat demand to price changes.

**Methods:**

This study used a cross-sectional survey design. Two stage sampling was used to recruit 350 khat chewers from four selected primary healthcare centers in Jazan province (South western province of Saudi Arabia). The data were collected during the first quarter of 2017. This study used both contingent valuation and revealed preference methods to assess the impact of price increases on the purchasing of khat. Graphical analysis, paired-samples t-test, and one-way repeated measures analysis of variance (ANOVA) were used to assess the impact of price increases on khat consumption.

**Results:**

The study results showed a significant decrease in khat consumption amount (t = 8.63, *p* ≤ 0.05), frequency (t = 30.42, *p* ≤ 0.05), and expenditure (t = 34.67, *p* ≤ 0.05) after the tightening of the Saudi–Yemeni border. Hence khat demand is price elastic. The price elasticity of khat demand in Jazan is estimated to be between − 2.38 and − 1.07. Therefore, each 1% increase in price is associated with 1–2% reduction in quantity demanded. This means khat chewers are relatively responsive to price changes (i.e., khat demand is price elastic). Repeated measures analysis of variance showed price increases significantly affect the quantity {F(4, 2.58) = 257, *p* ≤ 0.05, ηp^2^ = 0.423} and frequency {F(4, 1.83) = 415, *p* ≤ 0.05, ηp^2^ = 0.543} of khat chewing.

**Conclusions:**

Increased prices for khat would significantly decrease demand. Accordingly, we recommend implementing law enforcement strategies focused on disrupting the khat supply chain to realize high prices and so discourage use, hence reducing the incidence of khat-related illnesses.

## Background

Khat (*Catha edulis* Forsk) is a green plant that belongs to the family of Celesterece [1]. It is an amphetamine-like plant which is widely chewed in East Africa [2–7], Yemen [6, 8] and southern Saudi Arabia [9–14]. The main addictive substances in khat leaves are the cathinone and cathine (Schedule I and III drugs, respectively), which are prohibited by the United Nations’ International Convention on Psychotropic Substances [4, 15]. Khat leaves are usually culturally and socially chewed for various purposes such as enhancing social interaction, staying alert, decreasing appetite, inducing euphoria, improving mental performance prior to exams and increasing the self-esteem [16, 17].

However, different researches showed that khat chewing is associated with many health problems such as hypertension, gastrointestinal problems, inflammation of the esophagus and stomach, mouth ulcers, gum disease, coronary vasoconstriction and myocardial infarction [18]. It is also associated with increased vulnerability for stroke and early death. Khat consumption has attracted international concern as a result of spreading globally via migrants from khat endemic areas [4, 19–23]. Many countries have banned khat consumption due to its harmful medical and socioeconomic impacts [24]. The UK and the Netherlands are the two most recent countries to have enacted khat bans [25–28]. The Kingdom of Saudi Arabia has criminalized the cultivation and use of khat since 1957 [29].

**Fig. 1:**
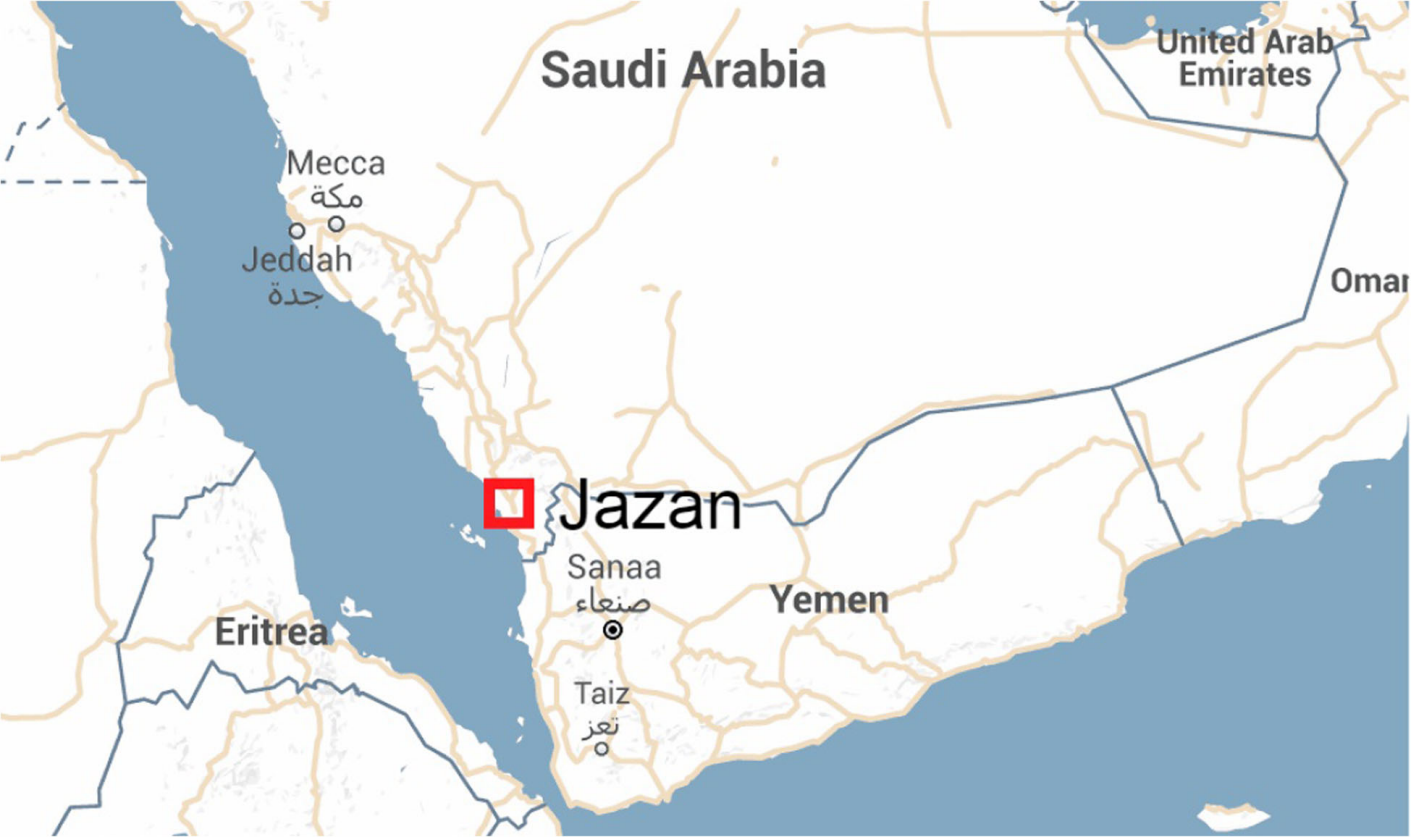
Jazan Region border with Yemen

Jazan lies in the far south-west of Saudi Arabia on its border with Yemen (Fig. 1). Jazan has approximately 1.6 million inhabitants, scattered throughout around 4000 villages, towns and islands. The Saudi government considers khat use the most pressing problem in this region. Traditionally, most of Jazan men used to chew khat [9, 29]. Since the establishment of the Substance Abuse Research Center (SARC) at Jazan University in 2011, research on khat has increased considerably [30]. SARC’s research has highlighted the high prevalence of khat use among school and university students [11, 12]. Moreover, the center researchers clarified the determinants of khat use in Jazan and its dependency potential [14, 31, 32].

The border between Jazan and Yemen used to be one of the primary routes for drug smuggling into the Kingdom of Saudi Arabia and the main route for khat smuggling into the Kingdom. In April 2015 the government of Saudi Arabia enforced more tightening on the Saudi/Yemeni border which was positively reflected on reducing khat smuggling and hence increasing its prices.

Saudi government had provided a lot of demand reduction facilities (resources) to those who want to quit. Currently, the treatment of addicts to any substance is free of charge in governmental hospitals without affecting their professional careers either if they seek treatment by themselves or detected through screening process. Given these demand reduction efforts, still some people choose to be addict and not willing to seek treatment. Law enforcement and supply reduction strategies might increase the cost and reduce the availability of substance to these individuals. The law enforcement strategy of supply reduction, focused on disrupting the drug supply chain through customs control, border closure, and/or policing is essential in fighting drug use [33–35]. Such a law enforcement strategy usually increases drug prices, and hence differentially affects drug users dependent on the demand elasticity of the drug concerned.

The importance and benefits of supply reduction policies include: i) encouraging some drug users to quit, ii) driving some others to reduce their consumption, iii) preventing relapse among quitters, and iv) preventing initiation by some potential new users. Therefore, the combined strategy of demand reduction and some degree supply reduction strategy could be more effective than each individual strategy. Contingent evaluation is one of the commonly used techniques to determine how people will respond to law enforcement strategy of supply reduction. It is an economic method obtaining a consumer’s reaction to hypothetical change [36].

Growing evidence-based research showed that drug users are relatively responsive to price changes (price elastic). Such research recommends using border interdiction to restrict drug supply, increase prices and so reduce the use [37]. Demand for addictive goods such as cigarettes and drugs tend to be highly inelastic [38–40]. In contrast, demand tends to be elastic in the case of normal goods, such as automobiles, or in the case of luxury goods, such as gold or diamonds [41, 42]. The responsiveness khat demand to price change has not yet been studied, and hence we aimed to assess the impact of price increases resulting from border tightening on khat consumption. Additionally, we aimed to estimate the responsiveness of khat users for hypothesized increase in its price. Khat bunches were sold for 100–150 Saudi Riyals each before the border tightening (1 bunch = 1 kilogram and 1 US$ = 3.75SR). After the border tightening, the one bunch of khat reached 600 SR and sometimes more.

## Methodology

### Study design and participants

This cross-sectional study was conducted in Jazan, Saudi Arabia. The data were collected February and June, 2017. Two-stage sampling procedure was used to recruit 350 khat chewers from four selected primary healthcare centers in Jazan province. The first stage involved simple random sampling in which we selected four (out of 13) primary healthcare centers (PHC) in Jazan province. In the second stage we invited all male khat users (chewing khat at least once/week during the last 4 years) to participate in the study. All users who voluntarily accepted to participate and to give a verbal consent for participation were involved.

**Fig. 2:**
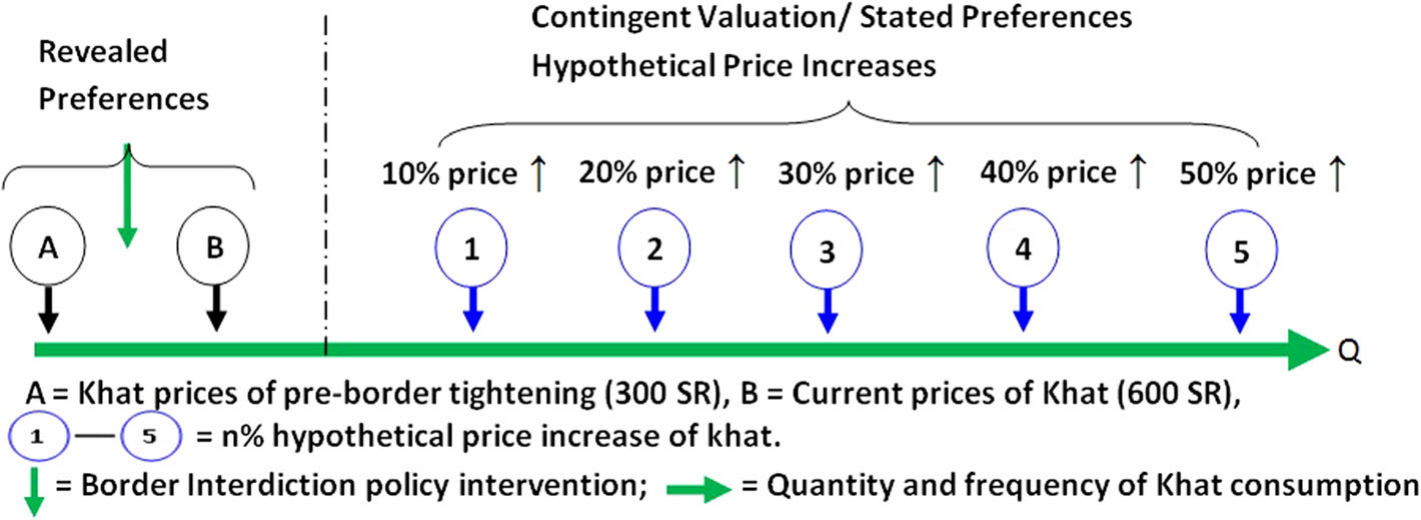
The protocol of the study

Figure 2 provides the research framework of the study. It shows that we used both contingent valuation (stated preferences) method and revealed preference method to assess the impact of price increases on the khat purchasing. Each participant was questioned about his actual khat consumption patterns before and after the border tightening (Point A and B). Moreover, they were questioned about the effect of hypothetical price increase (contingent valuation) on the quantity and frequency of khat consumption (point 1 to 5).

### Instrument and study protocol

We developed a two sections questionnaire. The first section consisted of questions on the preferences of actual khat consumption patterns before and after the border tightening. In this section participants were asked about their daily khat consumption, measured in punches, both before the Saudi–Yemeni border tightening and during the past 30 days. The variables studied in this section include; expenditure on khat consumption, quantity of khat consumption, and frequency of khat consumption. The second part was made up of contingent valuation (hypothetical) questions on the effect of price increases {at five different levels, namely 10% (660 SR), 20% (720 SR), 30% (780 SR), 40% (840 SR) and 50% (900 SR)} on the quantity and frequency and quantity of khat consumption. The question items in the questionnaire were validated by 5 experts in the field of health economics, public health, psychiatric and addiction. The hypothetical situations are listed in Table 1.

**Table 1:** Hypothetical Situations (Contingent Valuation/Stated Preferences questions)

Situation 1:	In case the price of Khat increased by 10% (1 bunch = 660 SR). How many punches of Khat will you chew per day?	In case the price of Khat increased by 10% (1 bunch = 660 SR). How many days will you chew Khat per day?
Situation 2:	In case the price of Khat increased by 10% (1 bunch = 720 SR). How many punches of Khat will you chew per day?	In case the price of Khat increased by 10% (1 bunch = 720 SR). How many days will you chew Khat per day?
Situation 3:	In case the price of Khat increased by 10% (1 bunch = 780 SR). How many punches of Khat will you chew per day?	In case the price of Khat increased by 10% (1 bunch = 780 SR). How many days will you chew Khat per day?
Situation 4:	In case the price of Khat increased by 10% (1 bunch = 840 SR). How many punches of Khat will you chew per day?	In case the price of Khat increased by 10% (1 bunch = 840 SR). How many days will you chew Khat per day?
Situation 5:	In case the price of Khat increased by 10% (1 bunch = 900 SR). How many punches of Khat will you chew per day.	In case the price of Khat increased by 10% (1 bunch = 900 SR). How many days will you chew Khat per day?

All data were collected by self-report in private interviews. Self-report has been found to result in sufficiently valid data. Darke (44) found self-reports to be valid when compared to biomarkers, collateral interviews, and criminal records, and concluded that self-reports result in accurate descriptions of drug use, related problems, and the history of drug use. Similarly, in a large-scale study, Denis et al. [43] found self-reported drug use on the ASI in an outpatient treatment setting to be accurate when compared to urinalysis results.

### Khat chewing index

We developed a simple khat chewing index (KCI) to quantify khat use. This index helped us categorize khat users into groups. This idea was based on the smoking index, which we modified to measure khat use.

*Khat chewing index (KCI) = Daily amount of khat use (measured in bunch, each roughly equivalent to one kilogram; these being a typical measure used to buy khat in Jazan area) × Usual number of days/week on which he used to chew khat during the last year × number of years of using khat.*

We then categorized khat users on a quartile basis to develop four categories. The first category (light users) comprised those users who scored below 25% on the chewing index. The second category (mild users) comprised users who scored in the range 25–50%. The third category (moderate users) comprised users who scored in the range 50–75%. The last category (heavy users) comprised users with chewing index scores that exceeded 75%.

### Measuring Price elasticity of demand

**Fig. 3:**
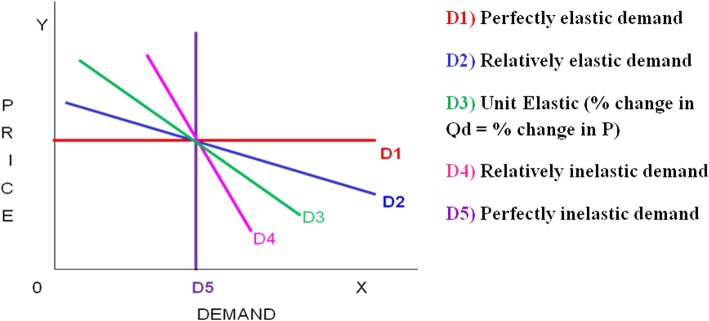
Different responses of demand to price changes

Price elasticity of demand (PED) can be defined as the responsiveness of the volume of demand for a good to a change in its price, when all other factors remain constant. Price elasticity is always negative because a negative relationship exists between price and volume demand (Fig. 3).

$$ {E}_d=\frac{\%\kern0.3em Change\ in\ quantity\ demanded}{\%\kern0.3em change\ in\ price}=\frac{\%\Delta  Q/Q}{\%\Delta  P/P} $$

PED can be assigned to the following three main categories:

1. PED > 1: this occurs when the proportional change in quantity exceeds the proportional change in price. That means demand is sensitive to price changes owing to consumers being price sensitive (Fig. 3: line D2).
2. PED < 1: this occurs when the proportional change in quantity is smaller than the proportional change in price. That means demand is not sensitive to price changes, owing to consumers being relatively price insensitive (Fig. 3: line D4).
3. Unitary elastic demand = 1: this occurs when the percentage change in volume demand equals the percentage change in price, a phenomenon that occurs rarely (Fig. 3: line D3).

### Statistical analysis

We analyzed all collected questionnaire data using SPSS (Statistical Package for Social Sciences) version 20. The data were analyzed systematically using descriptive and inferential statistics. We reported descriptive statistics using graphs with percentages, frequencies, means, and standard deviations. A paired-samples t-test was conducted to test the mean scores of ‘quantity of khat consumption’, ‘frequency of khat consumption’, and ‘expenditure on khat’ before and after border tightening. Then we used one-way repeated measures ANOVA to find the effect of hypothetical price increases on quantity and frequency of khat consumption. We tested the within-subject effects of different khat price levels on khat consumption. Hypothetical price changes were measured successively at five different hypothetical price increase points (10, 20, 30, 40, and 50%).

## Results

Socio-demographic characteristics of the respondents are shown in Table 2. Of the analyzed sample (*n* = 350), more than half of the respondents were married (63.9% of sample), while 11.1% were not married. More than four-fifths (76.5%) of the respondents were working in the private sector (82.0%), while the unemployed, retirees and government employees were less represented (8.9, 7.7, and 1.4%) respectively. More than half of the study participants (63.1%) were rural residents. About three-fourths (72.3%) of the respondents had completed post-secondary diploma level of education, followed by 24.3% who had secondary level education. Few respondents had primary school education (2.3%) and degree (1.1%). We had an overall 12% rejection rate (for different reasons) across the entire four primary healthcare centers and there was no difference in their rejection rate.

**Table 2:** Socio-demographic characteristics of the study participants

	Frequency	Percent
Marital Status		
Not Married	39	11.1
Married	311	88.9
Total	350	100
Education		
Primary School	8	2.3
Secondary School	85	24.3
Diploma	253	72.3
Degree	4	1.1
Total	350	100
Residence		
Urban	129	36.9
Rural	221	63.1
Total	350	100
Work Status		
Government	5	1.4
Private Sector	287	82.0
Retired	27	7.7
Unemployed	31	8.9
Total	350	100
Age		
18–24	21	6.0
25–34	101	28.9
35–44	97	27.7
> 45	131	37.4
Total	350	100.0

We studied the impact of price increases on khat demand using both revealed preferences and stated preference (contingent valuation) data. Table 3 shows the response to price changes of the mean quantity of daily khat demand. Before the tightening of the Saudi–Yemeni border average daily khat consumption was 813.6 g (95% CI 787.54, 839.60). After the border tightening the average daily khat consumption decreased to 720.0 g (95% CI 692.11, 747.89). This is equivalent to an 11.5% decrease in the average amount of daily khat consumption. Based on hypothetical data, a 10% increase in khat prices relative to current levels would decrease daily khat consumption to 547.1 g (95% CI 527.07, 576.21), equivalent to a decline of 32.76%. Given a price increase of 50%, daily khat consumption would decrease to 333.6 g (95% CI 315.67, 351.47), equivalent to a 59% decrease (Table 3).

**Table 3:** Mean daily khat demand volume in response to different price levels

Intervention	Mean quantity in grams (95% CI)*	Percentage decrease
Historical prices (150 SR)	813.6 (787.54, 839.60)	0.00
Current prices (600 SR)	720.0 (692.11, 747.89)	11.50
10% price increase (660 SR)	547.1 (527.07, 576.21)	32.76
20% price increase (720 SR)	537.9 (518.42, 557.30)	33.89
30% price increase (780 SR)	457.1 (439.47, 474.81)	43.82
40% price increase (840 SR)	371.4 (351.76, 391.10)	54.35
50% price increase (900 SR)	333.6 (315.67, 351.47)	59.00

One bunch is 1000 g (one kilogram), 1 U$ = 3.75SR

Note * = *p* < .001

**Fig. 4:**
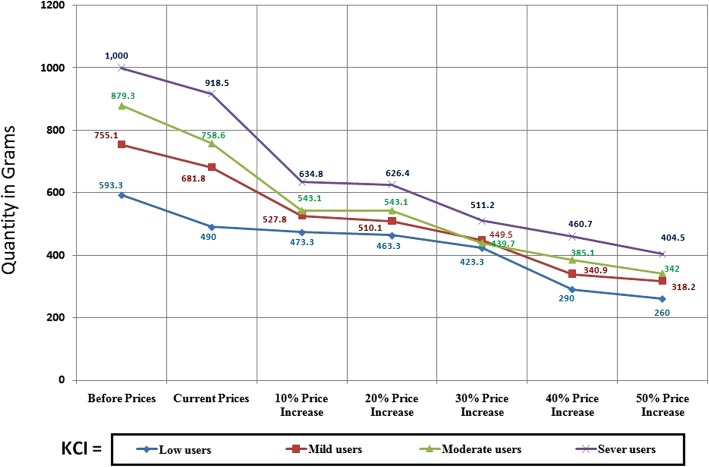
Changes in quantity of daily khat consumption in response to price increases among different categories of the KCI

The effects of the price increase on the quantity of khat use among different kaht chewing index (KCI) categories of our participants are shown in Fig. 4. Khat demand declined in response to price increases in all categories. The gaps in khat demand was wider among the indices of different khat users for the real data (revealed preferences data). In the case of the contingent valuation data, the gaps narrowed with further price increase.

**Fig. 5:**
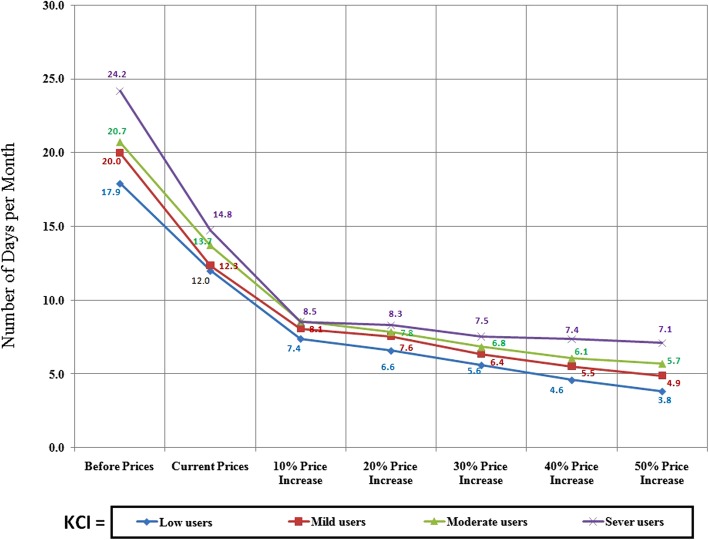
Changes in the frequency of the monthly khat use in response to price increases among different categories of KCI

Regarding the frequency of khat use, we found sharp declines in frequency in response to the first price increase (actual increase) among all categories of khat users (Fig. 5). The responses to the hypothetical increases were much smaller. Before the border tightening, heavy khat users reported chewing 24 times/month. This frequency dropped to 15 times / month after the border tightening. Usage frequency declined further to 8.5 times monthly with a hypothetical additional 10% increase in price. However, the response to further price increases then became much smaller. Moreover, this pattern of declining responsiveness to further price increases was consistent across other categories of khat users (Fig. 5).

**Fig. 6:**
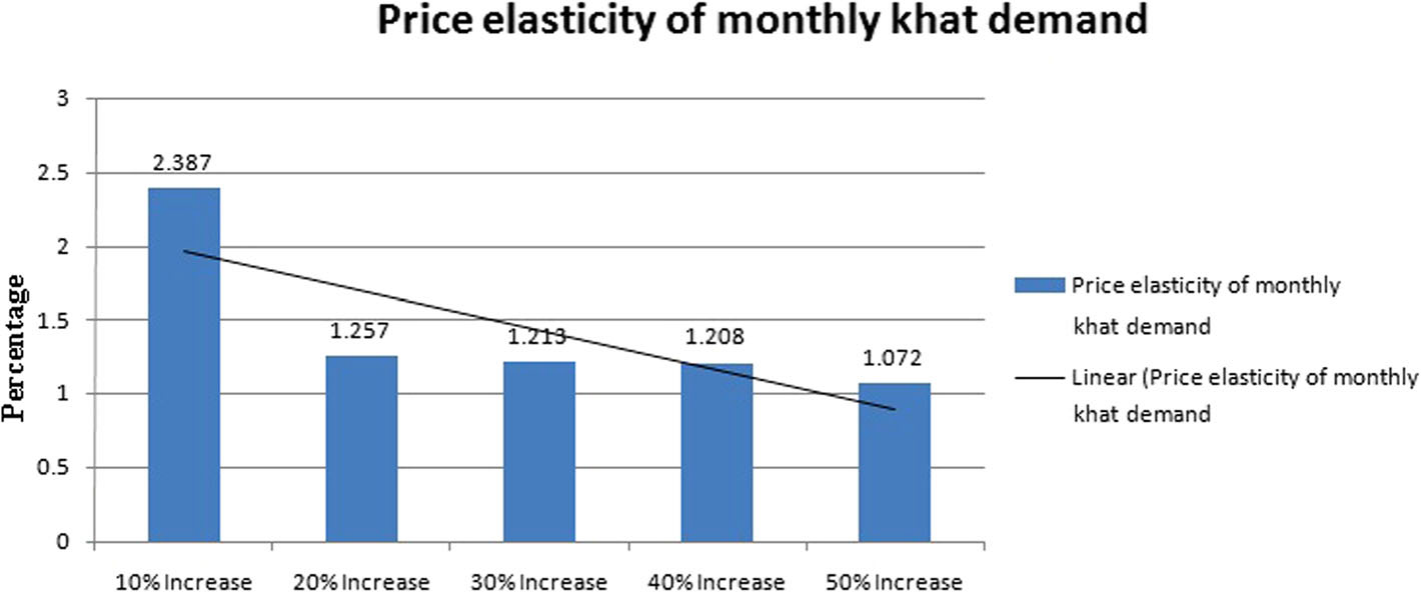
The price elasticity of monthly khat demand for different price increases

Figure 6 shows the price elasticity of monthly khat demand given different price changes. The price elasticity of khat demand in Jazan is estimated to be between − 2.38 and − 1.07 (price elastic). The elasticity decreases as the price of khat increases, with a sharp decline in response to initial smaller price increases flattening in response to subsequent larger increases. The price elasticity is largest at a 10% price increase, and smallest at a 50% price increase.

### Paired samples t-test

A paired-samples t-test was used to compare khat consumption quantity, frequency and khat expenditure on khat use before and after the tightening of the Saudi-Yemeni border. The Paired t-test showed significant decreases in consumption quantity {t = 8.63, *p* ≤ 0.05} and frequency {t = 30.42, *p* ≤ 0.05} after the border tightening. In contrast, a significant increase was detected in consumption expenditure {t = 34.67, *p* ≤ 0.05}. Before the border tightening, study participants spent a monthly average of Saudi Riyal (SR) 2238 on khat, which increased to SR 4345 after the border tightening (Table 4).

**Table 4:** Khat consumption quantity, frequency and expenditure before and after the border tightening

Items	Group Means (SD)	Mean differ. (SD)	95% CI		*P*-value
			Lower	Upper	
Khat consumption quantity (*in grams*):					
"Before the border tightening" vs	814 (247)	94 (20)	72	115	.001
"After the border tightening (Current)"	720 (265)				
Khat consumption frequency (*in # days*):					
"Before the border tightening" vs	21 (4)	7 (4)	6	8	.001
"After the border tightening (Current)"	13 (3)				
Expenditure on khat consumption (*in Riyals*):					
"Before the border tightening" vs	2239 (856)	2107 (1136)	2226	1987	.001
"After the border tightening (Current)"	4345 (1675)				

### Repeated measures analysis (ANOVA)

Repeated measures ANOVA testing was used to analyze the hypothetical responses of khat consumption to price increases (10, 20, 30, 40, and 50%). Mauchly’s test indicated that the covariance matrix assumption was not fulfilled; therefore, the F-values were corrected using the Greenhouse–Geisser test. The ‘Within-subjects test’ detected a significant effect of price increases on ‘khat consumption quantity’ (F = 256, df1 = 4, df2 = 2.585, *p* > 0.05, partial eta-squared = 0.423), and ‘khat consumption frequency’ (F = 415, df1 = 4, df2 = 1.834, *p* > 0.05, partial eta-squared = 0.543) (Table 5). We asked our participants about their expenditure on khat in revealed preference (reality) to show how real changes in price would affect their expenditure on khat. For the hypothetical questions we realized that it will be difficult for them to calculate their expenditure on khat. That is why we did not ask expenditure questions for these data.

**Table 5:** Summary of repeated measures tests of within-subjects effects

Effect of price change in	Mauchly’s test		Greenhouse-Geisser		Partial Eta Squared
	W	*p*-value	F (df_intervention_, df_Error[intervention]_)	*p*-value	
Quantity of khat consumption	0.222	< 0.001	256 (2.58, 902.1)	< 0.001	0.423
Frequency of khat consumption	0.125	< 0.001	415 (1.83, 639.9)	< 0.001	0.543

## Discussion

In this study we assessed the impact of the Saudi-Yemeni border tightening on khat smuggling as reflected on khat consumption quantity, frequency and expenditure. We also studied the behavioral responses of khat users in Jazan to hypothetical further increases in khat prices. We found that increased khat prices were associated with decreased khat use in terms of both amount and frequency. Meanwhile, increased khat prices as a result of the border tightening nearly doubled the average expenditure of khat users on their habit (Table 4).

Repeated measurement analysis revealed that hypothetical price increases of 10, 20, 30, 40, and 50% were accompanied by significant reductions in quantity and frequency of khat consumption. The partial Eta Square indicated that 42.3% of the variability in quantity and 54.3% of the variability in frequency of khat consumption reflected percentage price increases. The PED of khat chewers was relatively responsive to price change. So, a 1% increase in khat prices is associated with 2.38% decreases in demand volume. Conversely, a 1% decrease in khat prices is associated with 2.38% increases in demand volume (Fig. 6).

To our knowledge, this is the first study to estimate price elasticity of demand for khat consumption. We compared the PED of khat to other drugs such as tobacco, alcohol, marijuana, cocaine, and heroin. Based on National Health Survey data for the US the PED for tobacco use was estimated to be − 0.89 [44]. Although the PED for tobacco was price inelastic among the general population (− 0.89), it was highly elastic among young adults, meaning price sensitivity is inversely related to age [38, 44]. A recent Turkish publication measured price elasticity of tobacco demand before and after the implementation of anti-tobacco polices and showed that price elasticity was low before implementation (− 0.4) and increased post-implementation (− 0.72) [45]. Providing another useful comparison, the price elasticity of heroin is reported to be − 0.9 to − 0.8 and that of cocaine is − 0.055 to − 0.36 [39]. In contrast, the price elasticity for marijuana was estimated to be − 0.418 [40]. Generally the elasticity of substance use differs among countries and over time [38–40, 44, 45]. This variability may reflect the different natures of demand among different countries and the use of different statistical models.

The substances mentioned above (tobacco, marijuana, heroin, and cocaine) show low price elasticity (i.e. they are price inelastic), and hence exhibit price elasticity similar to necessities such as foods and nonalcoholic beverages. Therefore, price increases were not effective in reducing consumption of these substances, as people continue to consume them regardless of price. In terms of reported price elasticities these substances differ from khat, as reported in this paper.

In terms of price elasticity khat resembles alcohol and luxury goods (e.g., gold, diamond, automobiles, and airline travel), which are easily postponed [41, 42]. Douglas, J. Y [46] used data collected by the American Chamber of Commerce to estimate the price elasticity of alcohol and found it to be − 1.24. Similarly, Leung S. F, and Phelps, C. E [47] reported the price elasticity for wine to be − 1.0, and that of spirits (hard liquor) to be − 1.5. The price elasticities of these substances (khat and alcohol) exceed one, indicating that price increases are an effective means of reducing consumption, especially among the youth [48].

In contrast, the price elasticities for necessities such as food and nonalcoholic beverages were reported to be in the range 0.27 to 0.81 [49]. Price elasticity varies among different categories of food and non-alcoholic beverages. Examples of the price elasticities of different categories are as follows: beef 0.75 (0.67, 0.83), poultry 0.68 (0.44, 0.92), fish 0.50 (0.30, 0.69), fruit 0.70 (0.41, 0.98), sweets/sugars 0.34 (0.14, 0.53), cereals 0.60 (0.43, 0.77), milk 0.59 (0.40, 0.79), juice 0.76 (0.55, 0.98), and soft drinks 0.79 (0.33, 1.24). Because such items are important, consumed daily, and have few substitutes, they usually show price inelastic behavior.

Based on the above findings, law enforcement policies targeting drug users may offer an effective tool for drug control [50]. Economic analysis provides useful information about how law enforcement policies may reduce both supply and demand for drugs and so influence drug use behavior. The literature showed that implementing law enforcement strategies focused on disruption of the drug supply chain maintains high prices and consequently discourages use. However, the idea that supply reduction strategy will be always successful might seem too optimistic. Moreover, it might not be true in other substances. Khat could be unique as it is a plant that grows naturally and/or cultivated in Yemen which is the single main route for khat smuggling into Saudi Arabia. Having one route would be a possible cause for a successful supply reduction strategy. In summary, the costs of law enforcement strategies that increase khat prices must be weighed against the benefits of reduced khat consumption.

## Conclusion

Khat demand is price elastic, meaning khat resembles a luxury goods more than an addictive substance. The results show there was a significant reduction in quantity and frequency of khat consumption after border interdiction. Based on the study finding, we can conclude that law enforcement and supply reduction strategy (e.g. further border tightening, crackdowns and policing) drive prices up and restricts the availability of khat. Perhaps this may encourage the quitting of khat and may also minimize the associated health and social problems related with khat. In summary, combined strategy of demand reduction and some degree supply reduction strategy could be more effective than each individual strategy.

### Study limitations

This study collected the data from khat chewers and did not include quitters. Since, we included khat chewers only we were unable to report the substitution effect of the border tightening. Another study should be designed to target the substitution effect and the quitters. In this study we collected survey data (contingent valuation/ stated preferences) as it was difficult to get revealed preference data (time series data). We did not account for the two-stage sampling techniques in our analyses and assumed a one-stage sampling strategy. Fund limitation did not allow us to increase the sample size to report the elasticity in relation to education, income, gender, etc.

## Abbreviations

CI: Confidence interval
KCI: Khat Chewing Index
KSA: Kingdom of Saudi Arabia
PED: Price elasticity of demand
PHC: Primary healthcare centers
REC: Research Ethics Committee
